# From Conventional Reattachment to Intentional Replantation: A Conservative Approach to Complicated Anterior Tooth Fractures

**DOI:** 10.7759/cureus.115451

**Published:** 2026-08-30

**Authors:** Yash Sinha, Navin Mishra, Sulaima Mehar, Md Jawed Iqbal

**Affiliations:** 1 Department of Conservative Dentistry and Endodontics, Post Graduate Institute of Dental Education and Research, Indira Gandhi Institute of Medical Sciences, Patna, IND; 2 Department of Oral and Maxillofacial Surgery, Post Graduate Institute of Dental Education and Research, Indira Gandhi Institute of Medical Sciences, Patna, IND

**Keywords:** adhesive dentistry, anterior tooth fracture, fibre-reinforced post, intentional replantation, tooth fragment reattachment, traumatic dental injuries

## Abstract

Traumatic fractures of anterior teeth may result in substantial loss of coronal tooth structure and present complex restorative and endodontic challenges. Reattachment of the original tooth fragment is a conservative treatment option when the fragment is available, intact, and adequately adapted to the remaining tooth structure. This case series describes three anterior tooth fractures managed using tooth-fragment reattachment with progressively more complex treatment approaches. Case 1 involved a horizontal fracture of the gingival third of a maxillary central incisor and was treated with root canal treatment, fibre-post reinforcement and fragment reattachment. Case 2 involved a maxillary lateral incisor with a fracture extending approximately 2 mm subgingivally and was managed with root canal treatment, fibre-post support, surgical access and fragment reattachment. Case 3 involved a fracture extending to the junction of the cervical and middle thirds of the root. Intentional replantation was undertaken to facilitate extraoral endodontic and restorative management, followed by fibre-post-supported fragment reattachment and Mineral Trioxide Aggregate (MTA) placement at the fracture site. All the cases demonstrated satisfactory outcomes during the documented follow-up period of six months. These cases demonstrate how treatment strategies for fractured anterior teeth can be individualised according to fracture location, extent, accessibility, fragment condition and patient preferences.

## Introduction

Traumatic dental injuries are common and frequently affect the anterior teeth. Maxillary incisors are particularly susceptible because of their position within the dental arch, and anterior crown fractures represent an important component of traumatic dental injury. Falls, sporting activities and road traffic accidents are among the recognised causes of traumatic injury to anterior teeth [1,2]. Although many traumatic injuries involve a single tooth, severe trauma can affect several teeth and may result in complex fractures requiring multidisciplinary management [2].

The management of a fractured anterior tooth depends on the fracture pattern, remaining tooth structure, pulpal and periodontal involvement, condition of the fragment, and patient expectations. When the fragment is available and fits accurately, reattachment offers a conservative approach that preserves natural enamel and dentine while maintaining the original morphology, surface texture, contour and colour. This is particularly valuable in anterior teeth, where subtle differences in contour, translucency and surface anatomy can affect aesthetics. Predictable outcomes, however, require careful assessment of the tooth and fragment, accurate adaptation, appropriate adhesive procedures, and consideration of the fracture’s relationship with the gingival and periodontal tissues [3,4].

Complicated crown fractures may involve pulpal exposure and may extend towards or beyond the gingival margin. When the fracture line is subgingival, adequate access for endodontic and restorative procedures may require additional surgical intervention. Gingival flap elevation, orthodontic extrusion or surgical crown lengthening may be considered depending on the anatomical circumstances [5].

At the other end of the spectrum, fractures extending into the root may be difficult to manage using conventional intraoral procedures. Intentional replantation involves deliberate removal of a tooth followed by its replacement into the original socket. It can provide direct extraoral access to areas that are otherwise difficult to reach and has been described as an option for selected challenging endodontic situations [6,7]. Appropriate case selection, atraumatic removal, minimisation of extraoral time, maintenance of a moist environment and careful handling of the root surface are important principles of the procedure [6].

The present case series describes three anterior tooth fractures managed using preservation of the patient's original tooth fragment. The cases represent progressively more complex clinical presentations: conventional fibre-post-supported fragment reattachment, fragment reattachment of a subgingival fracture using surgical access, and intentional replantation followed by endodontic treatment and fibre-post-supported fragment reattachment.

## Case presentation

Case 1

A 22-year-old male patient reported to the Department of Conservative Dentistry and Endodontics, Post Graduate Institute of Dental Education and Research, Indira Gandhi Institute of Medical Sciences (PGIDER, IGIMS) with a fractured maxillary right central incisor following a bike injury (Figure 1). The patient's medical history was unremarkable. Clinical and radiographic examination demonstrated a horizontal fracture involving the gingival third of the crown and classified as an Ellis class III fracture (Figures 1a, 1b).

**Figure 1:**
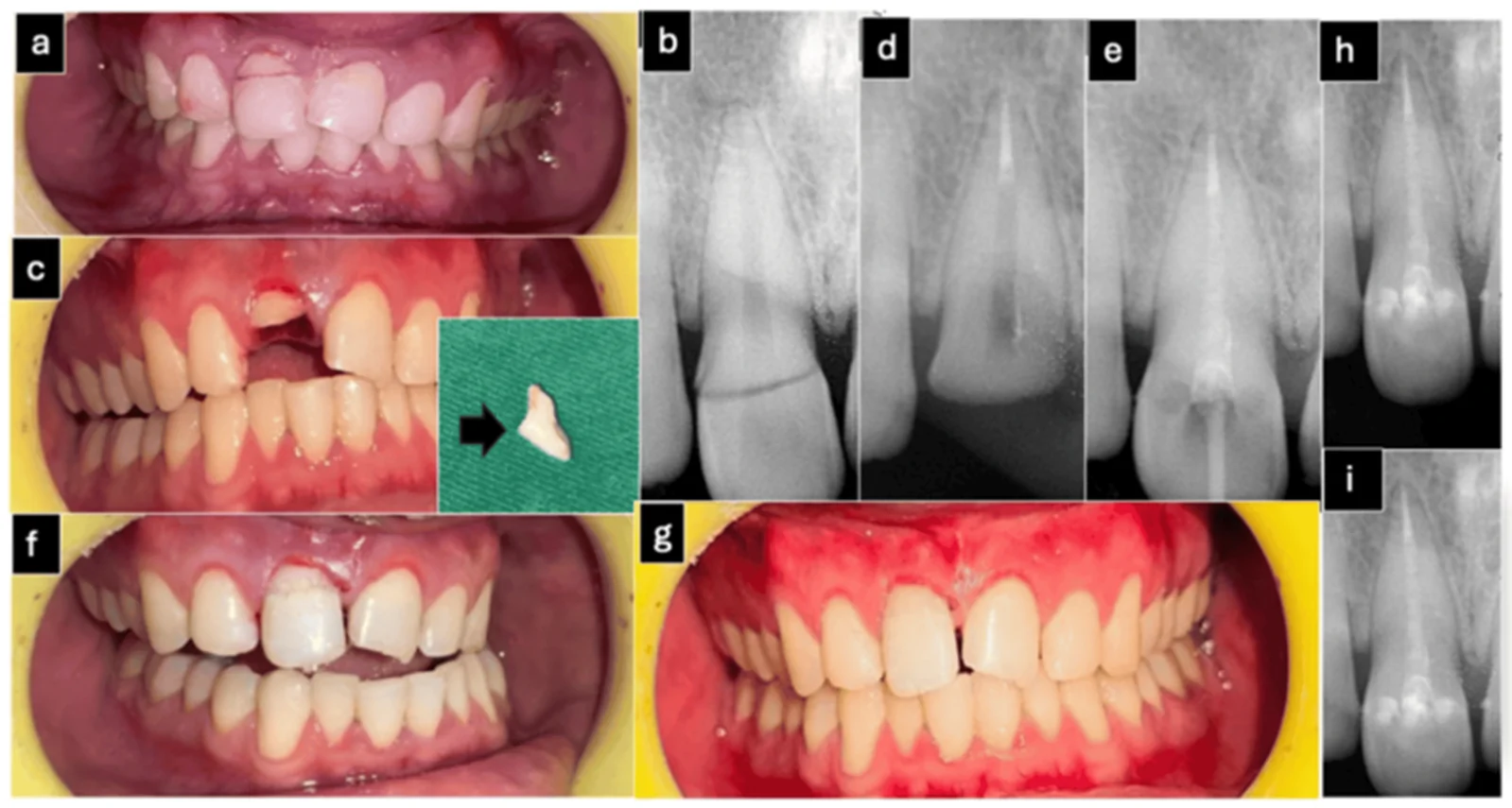
Clinical photographs of the case 1 patient during and after the treatment a) Pre-operative clinical photograph, b) Pre-operative radiograph, c) Clinical photograph after removal of fracture fragment (the black arrow shows the fractured coronal segment), d) Sectional obturation done in the apical 1/3rd of the fractured tooth portion, e) Radiograph after positioning of fiber post and coronal tooth fragment, f) Post-operative clinical photograph after repositioning of coronal tooth fragment, g) Immediate post-operative clinical photograph, h) Immediate post-operative radiograph, i) Post-operative radiograph showing six month follow up.

Two treatment options were discussed with the patient. The first involved removal of the fractured coronal portion followed by restoration using a post, core and crown. The second involved reattachment of the original tooth fragment. The advantages, disadvantages, prognosis and costs associated with the available treatment options were explained. The patient elected fragment reattachment. The fragment was considered suitable because it was in favourable condition and demonstrated adequate adaptation to the remaining tooth structure.

The fractured segment was carefully removed and thoroughly cleaned to eliminate debris (Figure 1c). It was maintained in sterile normal saline to minimise dehydration and preserve its integrity before reattachment. Root canal treatment was initiated promptly. Obturation was performed using a sectional technique while maintaining a 5 mm apical seal (Figure 1d). Following root canal treatment, post-space preparation was undertaken. A prefabricated glass-fibre post (Fibrapost PD, Switzerland) was selected and cemented into the canal using resin cement (Figure 1e). The fractured coronal fragment was subsequently prepared to receive the post and facilitate reattachment. A post space was created within the fragment, followed by etching with 37% phosphoric acid. After thorough rinsing, a bonding agent (Prime and Bond NT, Dentsply Sirona, USA) was applied.

Calibra resin cement (Dentsply Sirona, USA) was then placed within the post space and the prepared grooves in the coronal fragment. The fragment was accurately positioned against the remaining tooth structure and maintained under firm finger pressure during light curing (Figure 1f). Following polymerisation, excess restorative material was removed and the reattached area was finished and polished to obtain a smooth surface (Figures 1g, 1h). The patient underwent follow-up examinations at six-month intervals (Figure 1i). During the documented follow-up period, the tooth remained functionally and aesthetically satisfactory.

Case 2

A 23-year-old male patient reported to the Department of Conservative Dentistry and Endodontics, PGIDER, IGIMS with a complex crown fracture of the maxillary left lateral incisor following a traumatic fall (Figure 2). Clinical examination demonstrated a fracture extending from the cervical third of the crown on the palatal aspect to approximately 2 mm subgingivally on the labial aspect. The fracture was classified as Ellis class III (Figures 2a, 2b).

**Figure 2:**
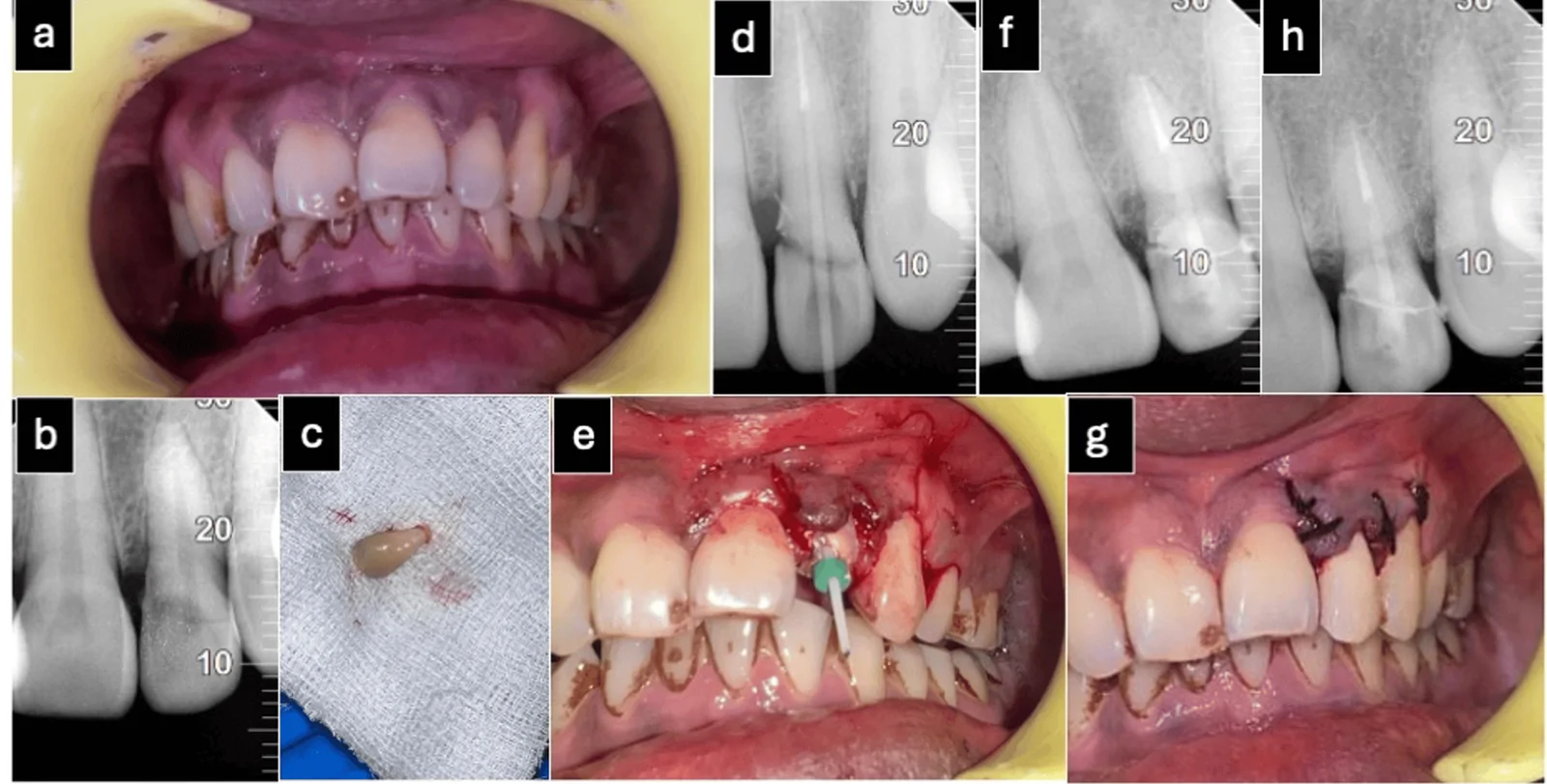
Clinical photographs of the case 2 patient before, during and after the treatment a) Pre-operative clinical photograph, b) Pre-operative radiograph, c) Clinical photograph after removal of fracture fragment (black arrow indicating fractured coronal segment), d) Radiograph after positioning of fiber post and coronal tooth fragment, e) Clinical photograph showing positioning of fiber post, f) Immediate post-operative radiograph, g) Immediate post-operative clinical photograph, h) Post-operative radiograph showing six month follow up.

Treatment options were discussed with the patient. The patient elected root canal treatment followed by reattachment of the fractured segment using a fibre post. Preservation of the natural tooth was an important consideration in the treatment decision. Under local anaesthesia, the fractured segment was carefully removed and maintained in normal saline to prevent dehydration (Figure 2c). Root canal treatment was initiated with coronal enlargement and root canal preparation. The canal was thoroughly irrigated using sodium hypochlorite and subsequently obturated with gutta-percha and endodontic sealer.

The subgingival extension of the fracture made direct access to the fracture line difficult. A gingival flap was therefore raised to provide access to the subgingival fracture and facilitate post placement. A prefabricated fibre post (Fibrapost PD, Switzerland) was selected, adjusted to the required length and cemented using dual-cured resin luting cement (Calibra, Dentsply Sirona, USA) (Figures 2d, 2e).

The coronal fragment was prepared by etching and bonding. It was then accurately repositioned and reattached to the remaining tooth structure using flowable composite resin (Esthet-X Flow, Dentsply Sirona, USA). Following completion of the restorative procedure, the gingival flap was repositioned and sutured. Occlusal adjustment was subsequently performed to ensure appropriate function (Figures 2f, 2g). At six-month postoperative clinical and radiographic evaluation, the tooth demonstrated normal function without clinical signs of distress. The documented clinical outcome was favourable (Figure 2h).

Case 3

A 31-year-old male patient reported to the Department of Conservative Dentistry and Endodontics, Department of Conservative Dentistry and Endodontics, Post Graduate Institute of Dental Education and Research, Indira Gandhi Institute of Medical Sciences (PGIDER, IGIMS) with a traumatic fall involving the maxillary right central incisor. The patient's medical history was unremarkable. Clinical examination revealed mobility of the coronal fragment (Figure 3). A preoperative periapical radiograph demonstrated a fracture extending to the junction of the cervical and middle thirds of the root (Figure 3a).

**Figure 3:**
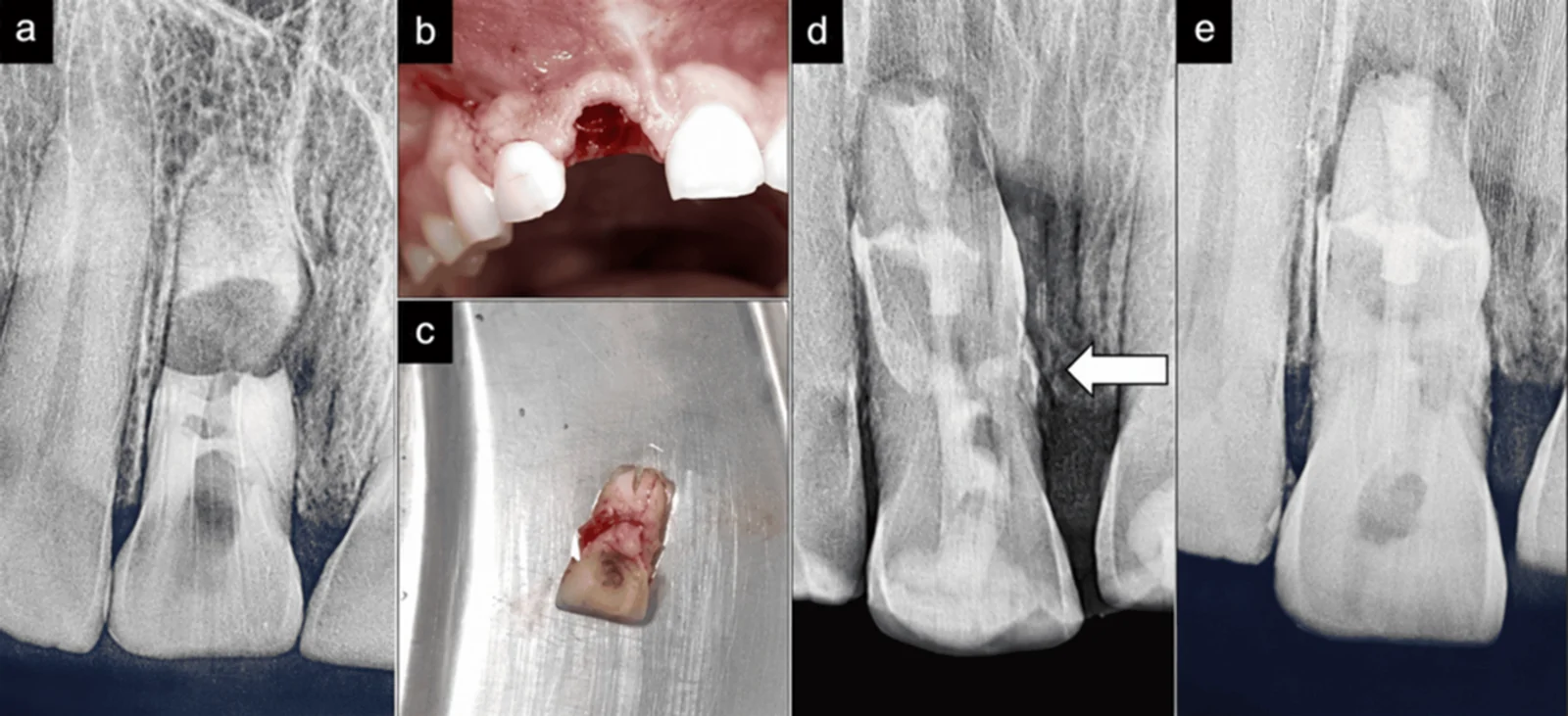
Clinical photographs of the case 3 patient before, during and after the treatment a) Pre-operative radiograph, b) Clinical photograph after removal of tooth, c) Clinical photograph showing separated coronal fractured fragment, d) Immediate post-operative radiograph (the white arrow indicates the placement of mineral trioxide aggregate (MTA) at fracture site,  e) Post-operative radiograph showing six month follow up.

Following clinical and radiographic assessment, treatment options were discussed with the patient. Because of the location of the fracture and the objective of preserving the natural tooth, intentional replantation followed by endodontic and restorative management was selected. Local anaesthesia was administered and the tooth was carefully removed using forceps with minimal pressure, with particular attention to atraumatic handling. The extracted tooth was immediately maintained in sterile saline (Figures 3b, 3c). The total extraoral treatment time was approximately 12 minutes. During the extraoral procedure, the root surface was handled carefully and cleaned as part of the treatment protocol. The socket was subsequently irrigated with saline.

Extraoral management was undertaken to provide direct access to the fracture site. Root canal treatment was performed, followed by preparation and placement of a fibre post using the post-space preparation and cementation principles described for Case 1. The fractured segment was subsequently repositioned and reattached using the fibre-post-supported technique. MTA (Pro-root MTA, Dentsply Mallifer, USA) was placed at the fracture site as part of the restorative management.

Following completion of the extraoral procedure, the tooth was repositioned into its original socket. The position of the replanted tooth was verified radiographically (Figure 3d). The treatment therefore combined intentional replantation, endodontic treatment, fibre-post reinforcement and MTA-assisted fragment reattachment. At the six-month follow-up, radiographic evaluation demonstrated satisfactory healing and stability of the replanted tooth (Figure 3e).

## Discussion

These three cases demonstrate different clinical presentations of anterior tooth fractures and illustrate how the treatment approach can be adapted according to the anatomical complexity of the fracture. Although the procedures differed considerably, the underlying treatment philosophy remained consistent: preservation of the patient's natural tooth and use of the original fractured fragment wherever clinically feasible.

The main advantage of fragment reattachment is preservation of the patient’s natural tooth structure and morphology. Using the original fragment maintains anatomical details such as surface texture, contour, incisal form and optical properties that may be difficult to reproduce with restorative materials [3,4]. However, the technique is not suitable for every case; the fragment should be available, sufficiently intact and capable of achieving good adaptation, while pulpal, periodontal, endodontic, coronal and occlusal conditions must be carefully evaluated before treatment [3,4].

Thus, conventional intraoral reattachment is most appropriate when the fracture line can be adequately accessed, and the fragment can be accurately repositioned and bonded. When the fracture extends subgingivally, additional procedures such as flap access, surgical crown lengthening or orthodontic extrusion may be considered according to the position of the fracture, periodontal requirements, remaining tooth structure and restorative objectives [8].

Case 1 represents the least complex end of this treatment spectrum. The fragment was available, sufficiently intact and adapted favourably to the remaining tooth, allowing conventional reattachment following endodontic treatment and fibre-post placement. Fibre posts are frequently used when additional intraradicular retention is required for the coronal restoration of structurally compromised endodontically treated teeth. Their clinical role should be interpreted primarily as providing retention and support for the restorative complex rather than as universally "reinforcing" the root. Evidence from systematic reviews suggests that fibre posts provide acceptable clinical performance in endodontically treated teeth, with some evidence of favourable fracture behaviour compared with rigid post systems, although differences in overall failure rates between fibre and metal posts are not consistently demonstrated [9,10]. In anterior teeth with substantial coronal tissue loss, a fibre post may therefore be a conservative means of retaining the reattached coronal fragment while maintaining an adhesive restorative approach. In the present cases, fibre posts were used specifically to provide intraradicular support for the reattached fragments.

The fracture in Case 2 presented a greater restorative challenge because it extended approximately 2 mm subgingivally on the labial aspect. In such circumstances, access to the fracture margin and maintenance of periodontal health become important determinants of treatment predictability. Surgical access by flap elevation was selected in this case to expose the fracture line and permit restorative management. Other approaches, including surgical crown lengthening or orthodontic extrusion, may be appropriate in selected subgingival crown-root fractures. Crown lengthening can expose sound tooth structure for restoration but may compromise periodontal architecture or aesthetics in the anterior region, whereas orthodontic extrusion can relocate the fracture margin coronally while preserving periodontal tissues in appropriately selected cases [8]. Consequently, treatment should be individualized rather than based solely on the depth of the fracture.

The outcome in Case 2 was favourable at the documented six-month clinical and radiographic assessment. However, this observation period should not be interpreted as evidence of long-term success. Clinical follow-up of reattached fragments should include assessment of fragment retention, marginal integrity, occlusion, periodontal health, mobility, colour stability and, where relevant, pulpal and periapical status. Clinical evidence has demonstrated satisfactory outcomes of fragment reattachment over long follow-up periods, but the available literature remains heterogeneous and is predominantly based on case reports, case series and small clinical studies [9,10]. Longer, standardized follow-up is therefore necessary before durable clinical success can be assumed.

Case 3 represented the greatest anatomical complexity, with a fracture extending to the junction of the cervical and middle thirds of the root and associated mobility of the coronal fragment. Conventional intraoral treatment would have provided limited access to the fracture line; therefore, intentional replantation was selected to provide direct extraoral access while retaining the natural tooth. Intentional replantation involves deliberate extraction of a tooth followed by treatment outside the socket and subsequent replacement into its original socket. Contemporary endodontic guidance considers intentional replantation a treatment option for selected situations in which conventional treatment or retreatment is impractical or inaccessible, provided that atraumatic extraction is feasible and the tooth has a reasonable periodontal and restorative prognosis [11]. It should not, however, be regarded as a routine substitute for conventional treatment.

The biological risks associated with intentional replantation also distinguish it from conventional fragment reattachment. The procedure requires manipulation of the tooth and periodontal ligament and may be associated with complications such as inflammatory root resorption and ankylosis. A systematic review and meta-analysis of intentional replantation reported a mean weighted survival of approximately 85.9%, with inflammatory root resorption and ankylosis reported among the principal complications [10]. Another systematic review reported a weighted mean survival of approximately 88% for intentionally replanted teeth, although root resorption remained an important complication [11]. These data suggest that intentional replantation can provide clinically useful tooth retention in appropriately selected cases, but its biological risks and the quality of the available evidence must be considered when comparing it with conventional reattachment.

The extraoral protocol in Case 3 was designed to minimize these risks. Atraumatic extraction, immediate placement in sterile saline, careful root-surface handling, an extraoral treatment time of approximately 12 minutes, and prompt replacement were undertaken. Contemporary guidance emphasizes atraumatic manipulation, preservation of periodontal ligament tissues, appropriate storage conditions and minimization of extra-alveolar time during intentional replantation [6,12]. The reported association between shorter extra-alveolar dry time and improved outcomes further supports minimizing the duration of extraoral manipulation [12]. The approximately 12-minute extraoral treatment period in the present case was therefore within the generally recommended short extraoral-time range. Nevertheless, the favourable six-month outcome should be interpreted cautiously because complications such as root resorption or ankylosis may become apparent only with longer observation.

MTA was placed at the fracture site in Case 3 as documented in the clinical procedure. However, because the available clinical information does not specify the precise indication, location, or thickness of the MTA, its therapeutic role should not be interpreted beyond the documented use. This distinction is important because the clinical indication for a calcium-silicate material should be explicitly reported when such materials are used in complex traumatic injuries.

The cases also emphasize the importance of the adhesive interface. In Case 1, the fragment was etched with 37% phosphoric acid, treated with a bonding agent, and reattached using resin cement, whereas Case 2 involved etching and bonding followed by flowable composite reattachment. There is currently no universally accepted adhesive protocol for fragment reattachment. A systematic review found substantial variation in adhesive systems and intermediate materials, including conventional and flowable composite resins and resin cements [13]. In vitro studies suggest that the reattachment interface can be modified by additional preparation of the fragment or remaining tooth. One study reported that internal dentine-groove and overcontour approaches produced greater fracture-strength recovery than simple reattachment, although the recovered strength remained below that of intact teeth [14]. The present cases therefore support meticulous adhesive preparation and accurate fragment adaptation, while recognizing that laboratory fracture resistance does not necessarily predict clinical longevity.

Patient preferences also influenced treatment planning. In Case 1, the patient chose reattachment over removal of the fractured portion followed by post, core and crown restoration after discussion of advantages, disadvantages, prognosis and costs. Preservation of the natural tooth was also important in Case 2. Thus, treatment selection should consider anatomical and biological factors alongside patient expectations.

The three cases demonstrate a progressive increase in treatment complexity. Case 1 involved a fracture within the gingival third and was managed with root canal treatment, fibre-post reinforcement and reattachment. Case 2 involved a subgingival fracture requiring surgical access and fibre-post-supported reattachment, whereas Case 3 involved a root-level fracture requiring intentional replantation for extraoral treatment. Therefore, fracture location and accessibility directly influenced the required intervention. Fragment reattachment may avoid unnecessary removal of remaining tooth structure when preservation is feasible. Although conventional post, core and crown restoration remains appropriate in selected cases, reattachment offers a conservative alternative when the original fragment is suitable for predictable retention.

This case series has several limitations. Only three patients were included; the cases were descriptive rather than comparative, fracture patterns and treatment approaches differed, and follow-up was only up to 6 months. Accordingly, these cases illustrate clinical treatment approaches rather than comparative effectiveness or long-term prognosis. Further long-term observation, particularly following intentional replantation, would be valuable. Assessment should include restoration integrity, periodontal status, mobility, pulpal and periapical findings where relevant, radiographic changes, and fragment stability.

Overall, these cases demonstrate that fragment reattachment can be adapted to varying fracture complexity, ranging from fibre-post-supported reattachment to approaches involving periodontal surgery or intentional replantation. Careful diagnosis, assessment of fragment suitability, atraumatic handling and appropriate patient selection remain fundamental to treatment planning.

## Conclusions

This case series demonstrates tooth-fragment reattachment as a conservative, tooth-preserving option for complicated anterior tooth fractures, with three progressively complex cases managed using the original fragments, including fibre-post-supported reattachment after endodontic treatment, surgical access for subgingival fracture, and intentional replantation with endodontic treatment, fibre-post reinforcement and MTA-assisted reattachment. Treatment was individualised according to fracture location and extent, accessibility, fragment condition and patient preference. When feasible, use of the original fragment can preserve natural tooth morphology and delay or avoid immediate prosthetic replacement. However, the limited and non-uniform follow-up restricts conclusions regarding long-term outcomes and emphasises the need for longer and consistently documented clinical follow-up.
